# Advances in Solar‐Driven Hygroscopic Water Harvesting

**DOI:** 10.1002/gch2.202000085

**Published:** 2020-12-13

**Authors:** Shendong Zhuang, Heshan Qi, Xueyang Wang, Xiuqiang Li, Kai Liu, Jun Liu, Han Zhang

**Affiliations:** ^1^ SZU‐NUS Collaborative Innovation Center for Optoelectronic Science & Technology International Collaborative Laboratory of 2D Materials for Optoelectronics Science and Technology of Ministry of Education Institute of Microscale Optoelectronics Shenzhen University Shenzhen 518060 China; ^2^ National Laboratory of Solid State Microstructures College of Engineering and Applied Sciences Jiangsu Key Laboratory of Artificial Functional Materials Nanjing University Nanjing 210093 China; ^3^ Department of Mechanical Engineering and Material Science Duke University Durham NC 27708 USA; ^4^ Institute of Advanced Technology Westlake Institute for Advanced Study Key Laboratory of Coastal Environment and Resources Research of Zhejiang Province School of Engineering Westlake University Hangzhou 310024 China

**Keywords:** collection, hygroscopic water harvesting, interfacial heating, solar‐driven steam generation, sorbents

## Abstract

Water scarcity is one of the greatest global challenges at this time. Significant efforts have been made to harvest water from the air, due to widely available water sources present in the atmosphere. Particularly, solar‐driven hygroscopic water harvesting based on the adsorption–desorption process has gained tremendous attention because of the abundance of solar energy in combination with substantial improvements in conversion efficiency enabled by advanced sorbents, improved photothermal materials, interfacial heating system designs, and thermal management in recent years. Here, recent developments in atmospheric water harvesting are discussed, with a focus on solar‐driven hygroscopic water harvesting. The diverse structural designs and engineering strategies that are being used to improve the rate of the water production, including the design principles for sorbents with high adsorption capacity, high‐efficiency light‐to‐heat conversion, optimization of thermal management, vapor condensation, and water collection, are also explored. The current challenges and future research opportunities are also discussed, providing a roadmap for the future development of solar‐driven hygroscopic water harvesting technology.

## Graphical abstract

Here, the recent developments in atmospheric water harvesting are reviewed, with a focus on solar‐driven hygroscopic water harvesting. Diverse structural design and engineering strategies for improving the rate of the water production are explored. The current challenges and future research opportunities for solar‐driven hygroscopic water harvesting technology are also discussed.

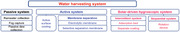

## 1 Introduction

### 1.1 The Status of the Earth's Water Resources

Water is the source of life. However, the shortage of freshwater resources has increasingly become a major challenge. Recent research found that about two‐thirds population of the world (about 4.0 billion people) live in a moderate water shortage for at least 1 month each year. And, more than 500 million people face extreme water shortages throughout a year. According to statistics, two‐thirds population of the world will face water shortages by 2025.^[^ ^1^, ^2^, ^3^, ^4^, ^5^, ^6^, ^7^, ^8^, ^9^, ^10^, ^11^, ^12^, ^13^, ^14^, ^15^, ^16^, ^17^, ^18^, ^19^, ^20^, ^21^, ^22^, ^23^ ^]^

#### 1.1.1 Limited Available Freshwater

Although 70.8% of the earth is covered by water, freshwater resources are extremely limited. According to data from the US Environmental Protection Agency, the total global water resources storage is ≈1.386 × 10^9^ km^3^, and freshwater resources account for only 3% of them. Regarding the distribution of freshwater, more than 68% is in the form of glaciers in the north and south poles or frozen soil, and the other 30% are buried deeply and dispersively as underground water. At present, freshwater resources (river, freshwater lake, and shallow underground water) that are more easily used by humans account for only 0.3% of total freshwater resources, which is equivalent to 0.007% of the global total water storage, about 1 × 10^5^ km^3^.^[^ ^4^ ^]^

#### 1.1.2 Unevenly Distributed Global Water Resources

At the same time, the distribution of global water resources is extremely uneven. According to statistics from the World Health Organization, the freshwater resources of the 9 countries with the most abundant water resources account for nearly 60% of all.^[^ ^1^, ^2^, ^3^, ^4^, ^5^, ^6^, ^7^, ^8^, ^9^, ^10^, ^11^, ^12^, ^13^, ^14^, ^15^, ^16^, ^17^, ^18^, ^19^, ^20^, ^21^, ^22^, ^23^ ^]^ In 80 countries and regions, which account for 40% population of the world, about 1.5 billion people are facing freshwater scarcity. Of these, about 300 million people in 26 countries even have extreme water shortages. Moreover, it is expected that by 2025, 3 billion people will face water scarcity in the world, and freshwater will be severely scarce in more than 40 countries and regions.

#### 1.1.3 Types of Water Scarcity

There are two types of water scarcity: 1) geographic water scarcity resulting from lack of direct water sources and 2) economical water scarcity resulting from weak economic and poor infrastructure that prevents the effective use of water resources.^[^ ^5^ ^]^ Typical geographic water‐scarce areas include Northern China, India, Middle East, Northern Africa, Western North America, Eastern Australia, etc. However, the average utilization efficiency of water resources can reach more than 75%. Typical economic water‐scarce areas are mainly on the African continent, due to its weak economic foundation and incomplete infrastructure, the average utilization efficiency of water resources is even less than 25%. Influenced by factors such as geography, climate, economics, population, and technology, the severity of water shortage is responded to variously in different regions of the world. How to solve the major challenge of water scarcity becomes an urgent task.

### 1.2 Conventional Water Treatment Technologies and Their Limits

In recent decades, in order to alleviate the shortage of freshwater resources, some water treatment technologies have been developed and applied.

#### 1.2.1 Seawater Desalination

In response to the lack of freshwater resources and the richness of seawater resources, people in some regions such as the Middle East, Arabia, North America, and Asia have begun to use seawater desalination technology to meet freshwater needs. Among the most common seawater desalination technologies are reverse osmosis, multieffect flash evaporation, vapor compression, electroosmosis, etc.^[^ ^6^, ^7^, ^8^, ^9^, ^10^, ^11^ ^]^ These technologies have effectively alleviated the shortage of freshwater resources in coastal areas.

#### 1.2.2 Waste and Sewage Treatments

For the multiple uses of water to reduce the scarcity of freshwater resources, waste and sewage treatment technologies have been proposed and continuously developed, mainly divided into physical methods, chemical methods, and biological methods.^[^ ^12^ ^]^ These technologies partially alleviate the shortage of freshwater resources. However, these technologies are more suitable for areas with direct water sources (visible water).

For geographic water‐scarce areas, such as Laos, Nepal, and Cambodia, the lack of water sources leads that it is impossible to use the abovementioned water treatment methods. As a result, a new solution, water harvesting from the air, has gained wide attention.

## 2 Atmospheric Water Harvesting

### 2.1 Water Resources in the Atmosphere

#### 2.1.1 Considerable Freshwater Resources in the Atmosphere

As an important part of the biosphere water cycle, a large amount of water vapor is stored in the atmosphere. Under the sun's irradiation, the water vapor evaporated from the sea surface is transported by the airflow to the interior of the continent. And as the altitude rises, the clouds gather into the rain. The rainwater converged on the river and flowed to the ocean again. As shown in **Table** **1**, the freshwater reserve of the atmosphere far exceeds that of swamps, wetlands, and rivers on the earth. It is estimated that the atmosphere contains more than 12.9 × 10^3^ km^3^ of freshwater, equivalently one‐eighth of the total freshwater resources of rivers and lakes.^[^ ^13^ ^]^ Therefore, the amount of water resources in the atmosphere, which is often overlooked, is actually substantial enough to meet the needs of human beings.

**Table 1:** Water and freshwater distribution data on earth.^[^ ^15^ ^]^

Water resources storage	Volume [× 10^3^ km^3^]	Proportion of total water	Proportion of freshwater
Brine reserves			
Ocean	1 338 000	96.54	0
Underground saltwater	12 870	0.93	0
Saltwater lake	85	0.006	0
Freshwater reserves			
Glacier	24 064	1.74	68.7
Underground freshwater	10 530	0.76	30.06
Permafrost	300	0.022	0.86
Freshwater lake	91	0.007	0.26
Soil	16.5	0.001	0.05
Atmospheric freshwater	12.9	0.001	0.04
Marsh/wetland	11.5	0.001	0.03
River	2.12	0.0002	0.006
Organisms	1.12	0.0001	0.003
Total water	1 386 000	100	
Freshwater	35 029	0.0025	100

The freshwater in the atmosphere is divided into three types according to its existing form: 1) clouds in the sky, 2) fog near the ground, and 3) water vapors in the air.^[^ ^14^ ^]^ Different from water vapor, both clouds and fog are made up of tiny water droplets with a diameter usually ranging from 1 to 40 µm (the diameter distribution of raindrops in rainwater is usually ranging from 0.5 to 5 mm).

### 2.2 Progress of Atmospheric Water Harvesting

#### 2.2.1 Early Water Harvesting Technologies

Water harvesting from air is old wisdom created by ancients. A Russian, Zibold, found that the early Greeks who established Theodosia in the sixth century BC might use dew condensation devices to capture water in the air, and conducted water harvesting experiments with a bowl‐shaped stone condenser.^[^ ^16^, ^17^ ^]^ Modern records in the first half of the 20th century, Zibold's experiment inspired Chaptal, Goddard, and Knapen to further modify this type of water condensing device in southern France. These Crimean‐like devices are called “air wells” or atmospheric water traps. Some of these devices successfully harvested the water in the air, but the water production was far below expectations. These huge water harvesting devices were made of stones, and the cold air at night made the stone pile reach a relatively low temperature. Then, during the daytime, the high‐humidity air from the ocean reaches the low‐temperature stone surface to be condensed to liquid water. However, due to the low thermal conductivity of the stone, the heat exchange performance of the entire system is poor, which severely limits the amount of water condensation. After the great hopes for this large‐scale “air well” device were broken, Monteith did a series of studies on dew condensation in 1957.^[^ ^18^ ^]^ He changed the focus from observing phenomena of dew condensation to understanding the energy conversion of dew formation and evaporation, as well as the heat balance mechanism. Since then, research on modern water‐harvesting devices has developed quickly,^[^ ^19^ ^]^ and a variety of different air and water collection methods have been developed,^[^ ^16^, ^20^ ^]^ most of which are used in arid or semiarid areas. In addition, compared to technologies such as desalination, when water is captured from the air, it does not affect the natural hydrological cycle. And, nature has a very strong recycling function, the amount of water lost will be continuously replenished. Therefore, harvesting water from the air could not disrupt the water balance in the atmosphere and not have a substantial adverse effect on the environment.^[^ ^21^ ^]^

#### 2.2.2 Modern Water Harvesting Technologies

**Figure 1:**
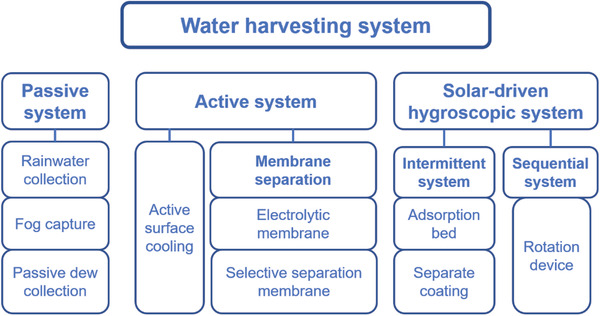
Classification of atmospheric water harvesting systems.

The research of modern water harvesting technologies is divided into three categories in detail according to the way of energy utilization (**Figure** **1**): 1) the traditional passive water harvesting system, in which this system can operate without additional input energy, 2) the active water harvesting systems that operate with additional input energy, i.e., electricity, 3) solar‐driven hygroscopic water harvesting that is rapidly developed in recent years. Water vapor is captured from the air by sorbents, and then the energy supported by solar energy drives the evaporation and desorption of water in sorbents, and at last the freshwater is collected. The following section will introduce in detail the typical systems of various types of water harvesting.

#### 2.2.3 Comparison of Water Harvesting Technologies

The advantages, challenges, and energy efficiencies of the current main water harvesting technologies are specifically analyzed and listed into **Table** **2**. In these water harvesting technologies, active surface cooling and membrane separation technologies require a large amount of electrical energy, and the devices are larger in size and complexity, but their water production is relatively larger. Passive surface cooling technology (radiative cooling) does not require energy consumption and the device is simple in construction. However, its water production is extremely small, and it is not universal. And, the solar‐powered hygroscopic water harvesting system has low cost, simple installation, comprehensive application scenarios, and input energy as solar energy. It is an environmentally friendly and effective water harvesting technology. It is expected that this interfacial solar‐driven atmospheric water generator, based on the liquid sorbent with a simultaneous adsorption–desorption process, opens up a promising pathway to harvest water from the air effectively.

**Table 2:** Analysis of atmospheric water harvesting technologies.^[^ ^22^ ^]^

Methods	Characteristics	Water yield	Application scenarios
Passive surface cooling^[^ ^25^ ^]^	Advantages: zero energy consumption, simple Disadvantage: a little water production	0.3–0.6 kg m^−2^ d^−1^	High dew point, good radiative cooling
Fog capture^[^ ^23^ ^]^	Advantages: low energy consumption, simple Challenges: unstable water harvesting, large footprint, maintenance	1.5–12 kg m^−2^ d^−1^	High dew point, high altitude
Active surface cooling^[^ ^24^ ^]^	Advantages: mature technology, continuous water harvesting Challenges: high energy consumption, high maintenance cost	9–18 kg m^−2^ d^−1^	High dew point
Membrane separation^[^ ^25^ ^]^	Advantages: good water quality, large water production Challenges: large energy consumption, immature technology, high cost	162 kWh m^−3^	Factory system
Solar‐driven system^[^ ^21^, ^26^, ^27^ ^]^	Advantages: solar‐driven, low dew point, portable Challenges: low water production, maintenance	1.0–2.89 kg m^−2^ d^−1^	Universal applications

### 2.3 Passive Water Harvesting

The traditional passive water harvesting system does not require external energy input to the system, and it can spontaneously harvest water in the air under different conditions. Because of its simple concept and low threshold for system installation, it was first developed and used by humans. It is generally divided into three types: 1) rainwater collection, 2) fog capture, and 3) passive surface cooling‐induced dew collection.

#### 2.3.1 Rainwater Collection

In ancient times, it was difficult to adjust the amount of water with the help of water conservancy and other projects. Therefore, the collection and utilization of rainwater have always been the top priority for household and agricultural water use. As early as 6000 years BC, the Aztec and Mayan cultural periods already had the practice of using rainwater, and people have applied rainwater to agricultural production and daily life. With the economic development and population growth, the water consumption of city is increasing, and the problem of water shortage has become more acute. This phenomenon has promoted the collection and use of rainwater.^[^ ^33^ ^]^

So far, the common rainwater collection methods include large‐scale projects such as building dams, surface runoff collection, barrage dams, ditches, etc. Building large‐scale water storage spaces to collect and store rainwater, with water treatment methods like filtering and disinfecting, are used in production in life. There are also miniaturized collection systems such as roof rainwater collection, groundwater tanks, rain buckets, etc., which are mostly used for family life.^[^ ^33^ ^]^

**Figure 2:**
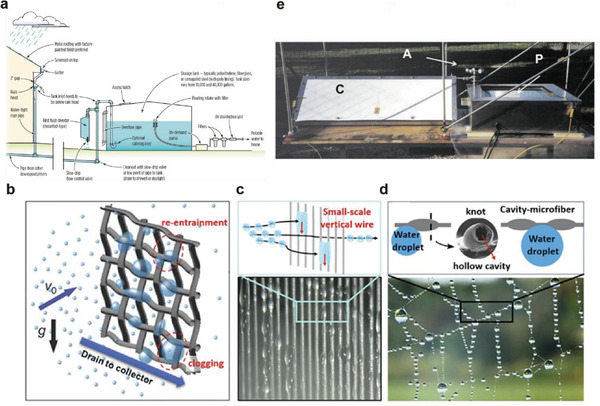
Passive water harvesting systems. a) Drinkable rainwater collection system. Reproduced with permission.^[^ ^28^ ^]^ Copyright 2020, Innovative Water Solution LLC. Fog capture system: b) traditional fine mesh fog capture structure, c) harp‐like fog capture structure, d) spider‐web‐like microfiber cavity topological fog capture structure. Reproduced with permission.^[^ ^21^ ^]^ Copyright 2018, Elsevier; Reproduced with permission.^[^ ^29^ ^]^ Copyright 2013, American Chemical Society; Reproduced with permission.^[^ ^30^ ^]^ Copyright 2018, American Chemical Society; and Reproduced with permission.^[^ ^31^ ^]^ Copyright 2017, Springer Nature. Dew collection system by passive radiative cooling: e) passive dew collection device by passive radiative cooling. Reproduced with permission.^[^ ^32^ ^]^ Copyright 2003, Elsevier.

Modern rainwater collection method for family is a potable rainwater collection system based on roof collection (**Figure** **2**a).^[^ ^28^ ^]^ The whole system is composed of several subcomponents such as water collecting area, initial flushing device, transportation pipeline, filter, pressure regulating pump/storage tank, and disinfection device. The rooftop collects the rainwater for the first time and transports it to the storage tank through the pipeline. The initial flushing device transfers the first wave of rainwater collected from the roof of the water tank for removing accumulated dust and debris over time. The first flush of rain will effectively take away these impurities. Then, the transportation pipeline network transfers water from the roof surface to storage containers and retransports the treated water. After filtering suspended particles or polluted impurities, the collected rainwater is transferred to water storage tank component and disinfected by the final disinfection device. The whole progress is driven by pressure regulating pump. The capacity of the water storage tank is enough for a family of three to use for 5–20 months.

#### 2.3.2 Fog Capture

Fog capture is a technically and practically feasible solution to the shortage of freshwater resources, especially in arid coastal areas. The usual way to collect fog is to build a rectangular net perpendicular to the wind direction to capture the water droplets in the fog. When the rectangular mesh device is placed in a fog environment, water droplets carried in the wind will hit the surface of the mesh and be captured. After a long period of continuous impact, the continuously captured water droplets will fuse and condensate and become larger. After a certain size, they will fall under the action of gravity and enter the collection tank through the transportation of the grid structure and the water tank.

In 1956, the Catholic University of North Antofagasta in northern Chile conducted the first experiment with a net cover.^[^ ^34^ ^]^ Since 1987, some similar but larger experiments have been carried out by Schemeenauer and co‐workers in many arid regions around the world, such as the coastal desert in West Africa (Namibia), South America (Chile and Peru), and the Middle East (Saudi Arabia and Oman, monsoon season).^[^ ^35^ ^]^ These successful experimental cases have inspired more similar projects to be launched and implemented around the world. The typical experimental climatic environment is that the droplet content in the air is 0.1–0.5 g m^−3^, 40% fog infiltration time, 3 m s^−1^ wind speed, and the collection efficiency is about 50%.^[^ ^36^ ^]^ Most of the advanced systems for high‐altitude fog capture have a water capture capacity of 3–7 kg m^−2^ d^−1^.^[^ ^36^ ^]^ In most previous projects, the fog capture device was not installed near the residence of the residents. Therefore, a pipeline needs to be installed to transport the captured liquid water to the residents in the mountains. The cost of the pipeline from the collection point to the residential homes is the main construction cost of this facility. Moreover, the entire system has high costs and difficulty in water transportation in practical use. Therefore, Abdul‐Wahab et al. studied the feasibility of constructing a fog capture device near a house residence.^[^ ^37^ ^]^ As described by Cereceda and co‐workers, a feasible and efficient fog capture device system should meet the following standards: 1) the frequency of fog in the climate of the area needs to be high enough and can be maintained for a long time, 2) fog with high altitude and high droplet water content is the main requirement for establishing a fog capture system in arid areas, 3) fog capture requires a certain wind speed to achieve higher collection efficiency.^[^ ^38^ ^]^

From now on, the biggest challenge is that the water collection efficiency of fog capture is too low. The efficiency is defined by the ratio of the amount of water actually collected in the water tank to the amount of water droplets perpendicular to the trap. The biggest problems with collection efficiency are currently two aspects that depend on surface wettability: reatomization of droplets that have been captured in the mesh and clogging of the collection mesh by fixed droplets (Figure 2b). The current metal network used for fog trapping has dual constraints: the coarse mesh structure cannot effectively capture tiny fog droplets, and the fine mesh structure is prone to droplet clogging. Although superhydrophobic surface treatment can prevent droplets from clogging to a certain extent, this superhydrophobic state cannot be maintained for a long time and cannot be used for a long time. Shi et al. proposed a new way to avoid these problems.^[^ ^30^ ^]^ They replaced the traditional cross‐network with small vertically arranged metal wires (harp‐shaped) (Figure 2c). Tian et al. proved that spider web structures assembled from low‐cost hollow fibers (Figure 2d) have high efficiency in directional water transport.^[^ ^31^ ^]^ Frankly, these methods show potential for improving collection efficiency, meanwhile, they still need to verify the real performance in more experimental scenarios.

#### 2.3.3 Passive Dew Collection

In water harvesting technology, dew collection is more effective than fog capture because it is less affected by climate and terrain, and it is also an ideal atmospheric water collection technology.^[^ ^20^, ^39^, ^40^, ^41^, ^42^ ^]^ Similarly, this technology is also a more cost‐effective alternative to artificial rainfall in areas with little cloud. At the beginning of the technology, the main research was on passive radiative cooling to collect dew,^[^ ^16^, ^20^, ^43^, ^44^ ^]^ because it does not require additional energy input. This type of radiatively cooled dew collection equipment usually works at night, because the daytime light will make the surface temperature of the condensing device almost equal to the ambient temperature. The main principle is to use the radiative cooling material of device to continuously radiate energy into deep space to reduce the temperature of the surface of the material and make it have a certain temperature difference from the ambient temperature. When the temperature difference reaches a certain value, the temperature difference drives the water vapor in the air to condense into liquid water on the surface of the low‐temperature radiative cooling material and then collects this part of the condensed water (Figure 2e). Researchers in Sweden and France^[^ ^14^, ^16^ ^]^ developed a set of applicable theoretical foundations to construct an effective radiative‐cooling‐based dew collection system. At present, the International Dew Collection and Utilization Organization has established a set of standard parameter settings in various aspects such as collection methods, equipment, and data in continuous experiments. The organization recommends the use of a special white low‐density polyethylene material as the standard collection material. The material has high hydrophilicity to reduce the nucleation barrier of water droplet condensation and has an extremely high emissivity in the infrared band of 7–14 µm. These two features are very important for radiation‐cooled dew collection materials. Based on the water vapor condensing capacity caused by the temperature difference that the radiative cooling material can produce, the researchers estimated the upper limit of dew production to be 0.8 kg m^−2^ d^−1^.^[^ ^39^ ^]^ However, in practical applications, the highest water production record in semiarid areas can only reach 0.3–0.6 kg m^−2^ d^−1^. Generally speaking, the condensation process is limited by the exchange rate of radiative heat, climatic conditions, and the surface properties of the condensing material. It is worth noting that especially weather conditions have a great influence on the heat exchange between the surface of the material and the air.

The applications of passive water harvesting technologies are limited by weather, climate, or geographical conditions, such as the impact of mountains and terrain, etc.

### 2.4 Active Water Harvesting

With the advancement of science and technology, the construction of an active water‐harvesting device through the application of energy, especially electric drive, enables the system to overcome objective restrictions, such as weather, climate, or geographical conditions, to achieve access to freshwater resources.

#### 2.4.1 Active Surface Cooling

With the development of the cooling machine, the active surface cooling device with additional energy input has gradually become the main way for the air to harvest water. Early active devices began to appear in the 1930s, but this technology has not been rapidly innovated and developed until the commercialization of mechanical refrigeration after 1980. Nowadays, active surface cooling water harvesting is regarded as an innovative technology that can manage the water supply of water quality/quantity in regional scope. Active surface cooling water harvesting technology is mainly divided into four types: 1) compression refrigeration, 2) electromagnetic cooling, 3) adsorption/absorption refrigeration, and 4) thermoelectric cooling.

##### Compression Cooling

**Figure 3:**
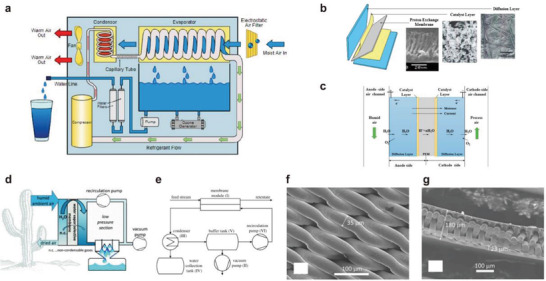
Active water harvesting systems. a) Schematic of compression cooling atmospheric water harvesting system. Reproduced with permission.^[^ ^22^ ^]^ Copyright 2013, Springer Nature. Electrolytic separation membrane system: b) structure and c) schematic of electrolytic separation membrane system. Reproduced with permission.^[^ ^45^ ^]^ Copyright 2017, Elsevier. Selective membrane permeation method of water vapor: d) the schematic diagram of the water vapor selective membrane method and e) the atmospheric water harvesting system. f,g) SEM images of selective permeability membrane. Reproduced with permission.^[^ ^46^ ^]^ Copyright 2015, Elsevier.

Compression cooling is the most widely used refrigeration technology for water harvesting.^[^ ^47^, ^48^ ^]^ As shown in **Figure** **3**a, with the help of the compressor, the liquid refrigerant is transported to the evaporating part, and the adsorbed humid air is quickly cooled to below the dew point, thereby condensing into liquid water. The low‐temperature cooled air is then used to remove excess heat from the condenser, and the obtained water is subsequently processed and stored.^[^ ^22^ ^]^ At present, nearly 90% of the water‐harvesting device products in the world use compression refrigeration, which covers domestic drinking water, industrial water, military applications, and even water supply irrigation. This type of product has applications ranging from small portable devices with an output of 20 L d^−1^ to large agricultural irrigation water‐harvesting devices with 200 000 L d^−1^.^[^ ^20^, ^49^ ^]^ The energy loss of this type of device mainly comes from the construction of the system such as the design of heat exchange components and device units. So far, the water consumption of this type of device is generally around 650–850 Wh kg^−1^ (electricity/water output), and the energy consumption efficiency of the most optimized device can even reach 250 Wh kg^−1^.^[^ ^50^ ^]^ The compression refrigeration device has the advantages of large water production and continuous water harvesting. However, due to insufficient airflow and heat exchange, it will cause problems such as frost on the surface, which will not only further reduce the heat transfer coefficient but also cause airflow resistance increases, which in turn increases energy consumption. Not only that, because the cycle requires refrigerant, such as Freon, etc., it will destroy the ozone layer.^[^ ^51^ ^]^

The other three cooling methods, namely electromagnetic cooling, adsorption/absorption cooling,^[^ ^52^ ^]^ and thermoelectric cooling are all unconventional cooling methods.

##### Electromagnetic Cooling

For electromagnetic cooling technology, the magnetic heat generation effect of a special solid refrigerant is used: the temperature of the refrigerant is controlled by adding and removing magnetic fields.^[^ ^53^ ^]^ This technology is usually environmentally friendly and pollution‐free, but the equipment is often heavy and expensive.^[^ ^54^ ^]^

##### Adsorption/Absorption Cooling

Adsorption/absorption cooling mainly relies on the conversion of electrical energy into heat energy as the driving force to achieve the reuse of absorption and desorption of hygroscopic materials. Because of the small number of components required, the circulating sorbents are usually ammonia, lithium chloride, calcium chloride, and other solutions. However, adsorption/absorption refrigeration devices are often bulky and expensive to manufacture.^[^ ^55^ ^]^

##### Thermoelectric Cooling

The thermoelectric cooling method is based on the principle of the Peltier effect of thermoelectric semiconductor materials:^[^ ^56^, ^57^ ^]^ When a thermocouple pair composed of two different semiconductor materials is fed with direct current, a temperature difference will be generated at the node of the thermocouple, and humid air will pass through the cooling end. Since the temperature of the cold end is lower than the dew point temperature, the water vapor in the air will condense into liquid water on the surface of the cold end to be collected. This technology‐type device is small and stable and requires no refrigerant. However, due to the poor performance of current materials, the overall energy conversion efficiency is low.

Active surface cooling technologies are relatively matured, meanwhile heavily dependent on the ambient temperature. For example, in dry air with a dew point temperature below 10 °C, their overall efficiency will be very low. And, these devices can hardly work when the ambient temperature drops to 4.68 °C.^[^ ^22^ ^]^

#### 2.4.2 Electricity‐Driven Membrane Separation for Water Harvesting

Membrane separation technology is an emerging technology in the field of water harvesting. In the past two decades, membrane separation technology has developed rapidly. It can be used in seawater desalination, sewage treatment, and other industries, and it is also of great industrial value in the field of water vapor separation. It is widely used in compressed gas drying,^[^ ^58^ ^]^ flue gas drying,^[^ ^59^ ^]^ natural gas drying,^[^ ^60^ ^]^ packaging materials,^[^ ^61^ ^]^ and clothing.^[^ ^62^ ^]^ The advantages are high energy utilization efficiency, low maintenance cost, simple components, and compact structure. Due to the different separation methods, membrane separation technology is mainly divided into two types: 1) electrolytic membrane separation method and 2) selective membrane permeation method.

##### Electrolytic Membrane Separation

The key component of the electrolytic separation method is a polymer electrolyte membrane (PEM), which is a five‐layer sandwich structure: a proton conductive membrane located in the middle, on both sides of which is a porous electrode with a catalytic layer and the outermost one with a diffusion layer porous electrode. As shown in Figure 3b, air flows through the pores of the diffusion layer of the cathode and anode, respectively. When direct current voltage is applied to the components, the following reactions occur at the anode and cathode of PEM:

Anode
  (1) $$\begin{array}{c} H_{2}O\;\;\to \;\;2H^{+}\;\;+\;\;2e^{-}\;\;+\;\;0.5O_{2} \end{array}$$
Cathode
  (2) $$\begin{array}{c} 2H^{+}\;\;+\;\;2e^{-}\;\;+\;\;0.5O_{2}\;\;\to \;\;H_{2}O \end{array}$$

The standard electrode potential for this reaction is 1.229 V. Under a higher electric field, the water vapor in the moist air of the anode is electrolyzed to produce H^+^ protons. The water molecules combined with these H^+^ protons move to the cathode under the drive of an electric field. At the same time, driven by the concentration, water molecules continue to move toward the anode where the reaction takes place. After that, the generated H^+^ will react with O_2_ at the cathode to generate new H_2_O. The newly generated water vapor will be taken away by the process gas and then condensed and collected (Figure 3c).

##### Selective Membrane Permeation

The selective permeation method separates water vapor from other gases in the air by a water vapor selective membrane. The driving force for penetration is the differential pressure across the selective membrane. This driving force passes through the steam condensing unit and a vacuum pump for pressurization. Due to the use of a dense polymer membrane with high selectivity for water vapor, almost no contaminants or pathogens can penetrate the membrane, making the water obtained by condensation very pure. In addition, the maintenance of the membrane will not be a major problem, because only the air containing water vapor is fed as the feed, and due to the lack of sunlight and water environment, there will be no bacteria and algae reproduction. This is also a very important advantage of this technology.

As shown in Figure 3d,e, the membrane module (I) exposed to the humid airflow (feed port) is the core of the system.^[^ ^25^ ^]^ And, Figure 3f,g shows the scanning electron microscope (SEM) images of selective permeability membrane. Due to its own selectivity and the pressure applied by the vacuum pump as the driving force, water vapor will pass through the membrane and the remaining dry air is removed. The driving force for the transmission of water vapor on the membrane is maintained by two main components: a vacuum pump (II) that controls the pressure on the permeate side and a heat pump (III) for condensing the selectively permeated water vapor. Therefore, the water vapor pressure showed in osmometer depends on the temperature of the condensation side and the corresponding saturated vapor pressure. The condensate is collected in the water collection tank (IV). And, a buffer tank (V) can be added to increase the volume of permeation measurement so that the whole system is not easy to be adversely affected by pressure changes. The circulation tank (VI) is used to create a low pressure so that the material circulation is accelerated and facilitates the separation of water vapor for osmometry.

The active water‐harvesting devices could overcome most restrictions from climate, weather, or geographical factors, but rely heavily on electricity, namely fossil fuels.

## 3 Solar‐Driven Hygroscopic Water Harvesting

**Figure 4:**
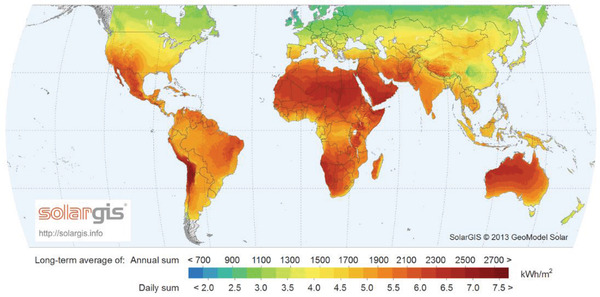
Solar energy distribution around the world. Global horizontal irradiation. Reproduced with permission.^[^ ^63^ ^]^ Copyright 2019, World Bank Group.

In order to reduce the dependence on fossil fuels, and slow down their damage to the environment, more and more researchers began to use solar energy as a driving force to harvest water from the air. It is mainly based on the advantages of solar energy: wide distribution (see **Figure** **4**) and nonpollution. Moreover, the water production efficiency is relatively high.

### 3.1 Working Principles of Solar‐Driven Hygroscopic Water Harvesting

#### 3.1.1 Work Process of Solar‐Driven Hygroscopic Water Harvesting

**Figure 5:**
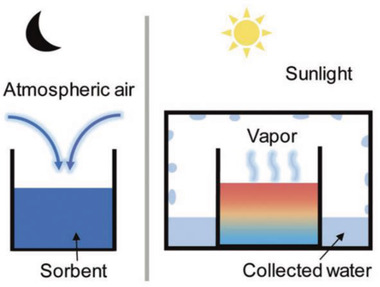
Schematic of solar‐driven hygroscopic water harvesting. Reproduced with permission.^[^ ^26^ ^]^ Copyright 2019, John Wiley and Sons.

The solar‐driven hygroscopic water harvesting technology is an ancient method, which uses sorbents to capture water molecules by physical or chemical adsorption from the air in the shadow, and then makes the water molecules in the sorbent desorption under the heating of sunlight, as illustrated in **Figure** **5**. The water vapor generated in this part will be recondensed, liquefied, and collected. The heated sorbents are recooled in the shadow for the next water vapor capture. The essence of water harvesting technology is to increase the partial pressure of condensed water vapor during the condensation collection process, thereby increasing the dew point temperature. Compared with the directly cooled water harvesting technology that the required condensation dew point temperature is lower, which is not conducive to the condensation of water vapor, the hygroscopic water harvesting technology can effectively improve energy utilization and increase the final water production.

#### 3.1.2 Adsorption and Desorption of Water Vapor

**Figure 6:**
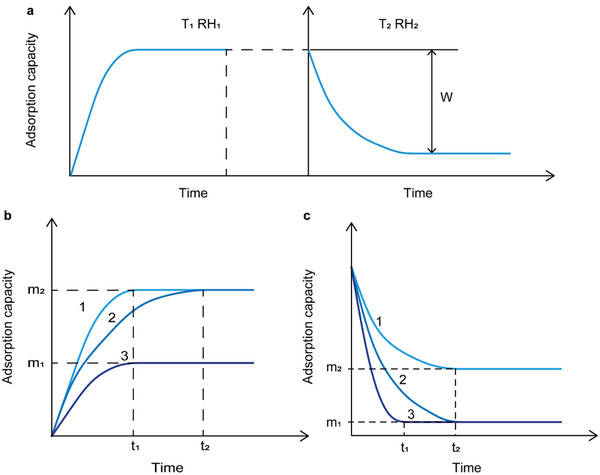
Schematic of adsorption and desorption processes. a) Dynamic diagram of adsorption and desorption. b) The dynamic diagram of adsorption of three types of sorbents. c) The dynamic diagram of desorption of three types of sorbents.

**Figure** **6**a shows a schematic of dynamic isothermal adsorption and desorption curves. During the operation of the water‐harvesting device, the theorical water production is the difference between its adsorbed water capacity (temperature *T*_1_ and relative humidity RH_1_) and the remaining water of the sorbents (temperature *T*_2_ and relative humidity RH_2_). And, the equilibrium theoretical water production is *W*.

In the process of harvesting water from the air, the water adsorption performance of the sorbents is expressed in the adsorption rate and adsorption capacity. As shown in Figure 6b, the amounts of saturated water adsorption of type 1 and type 2 sorbents (*m*_2_) are higher than that of type 3 (*m*_1_), which means that type 1 and type 2 have a relatively higher water adsorption capacity. And, the adsorption time *t*_1_ is less than the type 2 *t*_2_, which means that the type 1 sorbent has a faster absorption rate. The ideal water harvesting material often needs to have both the characteristics of water adsorption capacity and water adsorption rate.

Similarly, for the water desorption process of the sorbents, it is in terms of water desorption capacity and water desorption rate, as shown in Figure 6c. The type 3 sorbent is more excellent in desorption performance than that of the type 1 and type 2, because it has much higher amount of water in desorption process and less time to reach the equilibrium of water desorption.

#### 3.1.3 Condensation and Collection of Harvesting Water

When water in sorbents is desorbed and become water vapor, it needs to be condensed to collect. The condensation process requires specific temperature and relative humidity and pressure, which is dew point. In other words, when water molecules are condensed and nucleate from vapor to liquid state, if one of the three factors above changes, the other two will change at the same time. In general, the condensation process is a bottleneck of water harvesting due to two aspects: 1) the contradiction between the low desorption temperature of steam and high condensing temperature of water vapor – for desorption process, in order to improve the amount of steam generated, it is expected to lower the temperature of steam, and utilize the solar energy more to vaporize water molecules. However, for condensation process, higher temperature of steam will make it easier to nucleate of water molecules. These two contradictions result in difficulties; 2) stable low temperature of condensing surface – when generated steam contacts condensing surface, if the heat exchange of condensing area is not good, the temperature of this area will increase, which can hinder further condensation process. Therefore, condensation becomes a challenge in water harvesting.

#### 3.1.4 Energy Analysis from Harvesting to Collection

The energy conversion efficiency from sunlight to steam is the evaporation efficiency. The sunlight or the light emitted by the solar simulator directly irradiates the surface of the light absorber, and the real‐time monitoring of the mass change of the water body is used to calculate the mass flow that evaporates the water body. The efficiency (η) of the energy conversion process can be calculated using the Equation (3)^[^ ^64^, ^65^, ^66^ ^]^

(3) $$\begin{array}{c} \eta \;\;=\;\;\frac{m\left(L_{v}\;+\;Q\right)}{P_{\text{in}}} \end{array}$$

In the above Equation (3), *m* is the unit mass flow of the water body under light, which is equal to the difference between the mass flow of the evaporation system under light (*m*_light_) and under no light (*m*_dark_). *L*_v_ is the enthalpy of evaporation during the evaporation of water (different from pure water enthalpy, water molecules interact with sorbent molecules), *Q* is the sensible heat change of the water and sorbent (caused by the change in temperature), and*P*_in_ is the incident light power of sunlight.

In the conversion of sunlight to steam, in addition to the energy used for evaporation, other energy losses come from optical loss and thermal loss. The optical loss is due to the incomplete absorption of incident solar energy by the light absorber. Thermal loss includes three forms, namely: heat conduction, heat convection, and heat radiation. By analyzing the energy loss of the system, on the one hand, the system can be optimized, on the other hand, it can also be used to check the accuracy of the energy conversion efficiency of the system calculated by Equation (3).

The heat conduction process is the process of transferring energy from a high‐temperature object to a low‐temperature object through a substance when there is no macroscopic motion of the object. The heat flux density can be expressed as the following Equation (4)

(4) $$\begin{array}{c} q_{\text{cond}}\;\;=\;\;k\frac{T_{1}\;-\;T_{2}}{L} \end{array}$$

where *k* is the thermal conductivity coefficient, *T*_1_ and *T*_2_ are the temperature of the high temperature object and the low temperature object, respectively, and *L* is the thickness of the heat conduction medium.

Thermal convection refers to the phenomenon that heat propagates from one place to another through a flowing medium. The process of convective heat transfer between a constant temperature heat source and a fluid can be expressed as

(5) $$\begin{array}{c} q_{\text{conv}}\;\;=\;\;h\left(T\;-\;T_{c}\right) \end{array}$$

where *h* is the convection heat transfer coefficient, *T* is the temperature of the heat source, and *T*_c_ is the temperature of the surrounding fluid.

The phenomenon that an object radiates electromagnetic waves due to its temperature is called thermal radiation, and the object also absorbs radiation while emitting radiation. The net radiation of an object can be expressed by the Stefan–Boltzmann equation (Equation (6))

(6) $$\begin{array}{c} q_{\text{rad}}\;\;=\;\;\varepsilon \sigma \left(T^{4}\;-\;T_{c}^{4}\right) \end{array}$$

where ε is the emissivity of the object, σ is the Stefan–Boltzmann constant.

For the solar‐driven evaporation process, the entire energy transfer process can be expressed by the following Equation (7)

(7) $$\begin{array}{c} m\left(L_{v}\;+\;Q\right)\;\;=\;\;A\left(\alpha P_{\text{in}}\;\;-\;\;\left(q_{\text{cond}}\;+\;q_{\text{conv}}\;+\;q_{\text{rad}}\right)\right) \end{array}$$

where *A* is the projected area of the absorber.^[^ ^66^ ^]^

For the water vapor condensation process, the entire energy transfer process can be expressed by the following Equation (8)^[^ ^26^ ^]^

(8) $$\begin{array}{c} \eta \;\;=\;\;\frac{m_{2}\left(L_{\nu }\;\;+\;\;Q\right)\;\;-\;\;P_{\text{ex}}}{m_{1}\left(L_{\nu }\;\;+\;\;Q\right)} \end{array}$$

In Equation (8), *m*_1_ is the unit mass flow of the water body under light, which is equal to the difference between the mass flow of the evaporation system under light (*m*_light_) and under no light (*m*_dark_), *m*_2_ is the unit mass flow of the condensational water vapor, *L*_v_ is the enthalpy of evaporation during the evaporation of water (different from pure water enthalpy, water molecules interact with sorbent molecules), *Q* is the sensible heat change of the water and sorbent (caused by the change in temperature), and *P*_ex_ is the incident extra condensational energy.

### 3.2 Construction of Solar‐Driven Hygroscopic Water Harvesting System

Based on the solar‐driven hygroscopic water harvesting technology, the most important three factors of the system construction are: 1) the hygroscopic process of capturing water molecules from the air, 2) the desorption–evaporation process of the water in the sorbents, and 3) the condensation process of the generated water vapor. These three processes correspond to the selection of sorbents, the solar‐driven photothermal conversion, and the design of the condensation system.

#### 3.2.1 Sorbents

The selection of the sorbents is crucial in the entire hygroscopic water‐harvesting system. It directly determines the capture capacity of the entire device for water vapor and the upper limit of the final water production. The primary selection of sorbents is divided into three types: 1) solid sorbents, 2) liquid sorbents, and 3) composite sorbents.

##### Solid Sorbents

The solid sorbents are the earliest utilized and commercially available sorbents. They were first used for dehumidification of air, such as molecular sieve, silica gel, activated alumina, and zeolite. Molecular sieves are widely used in both closed adsorption and open dehumidification cooling systems.^[^ ^53^, ^54^, ^56^ ^]^ The molecular sieve can form a robust intermolecular force with water molecules with high stability. It needs to reach a high temperature in the process of water desorption. Its adsorption heat is about 3300–4200 kJ kg^−1^, so the molecular sieve is not suitable for the water harvesting system powered by solar energy.

The adsorption heat of silica gel particles is 2500 kJ kg^−1^, which is suitable for low‐temperature solar driving desorption. However, its equilibrium adsorption capacity is only 0.4 g g^−1^. If it is used in a water‐harvesting device, it needs to be filled with a large amount of silica gel, which will increase the mass and volume of the device and greatly reduce the efficiency of heat exchange.

**Figure 7:**
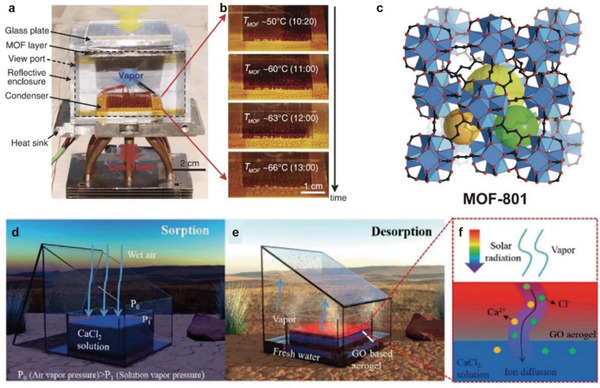
Conventional solid and liquid sorbents. a–c) MOF‐801 solid sorbent. Reproduced with permission.^[^ ^67^ ^]^ Copyright 2017, The American Association for the Advancement of Science. d–f) CaCl_2_ solution liquid sorbent. Reproduced with permission.^[^ ^27^ ^]^ Copyright 2019, John Wiley and Sons.

In recent years, many new solid sorbents such as inorganic nanoporous materials,^[^ ^68^, ^69^ ^]^ aluminum phosphate,^[^ ^70^ ^]^ metal–organic framework (MOF) materials^[^ ^67^ ^]^ have been continuously developed and utilized. Kim et al. used MOF‐801 to achieve a water output of 1.2 kg m^−2^ d^−1^ at a relative humidity of 20–30% (**Figure** **7**a–c).^[^ ^67^ ^]^ Novel nanomaterials have shown great potential in water harvesting with good water adsorption and water production.

##### Liquid Sorbents

Liquid sorbents need high hygroscopicity because the saturated vapor pressure of the solution is low. There are many types of liquid sorbents, such as 1) highly concentrated salt solutions: calcium chloride (CaCl_2_) solution,^[^ ^27^ ^]^ lithium chloride (LiCl) solution,^[^ ^71^ ^]^ and lithium bromide (LiBr) solution, and 2) high‐purity organic solutions such as triethylene glycol solution.^[^ ^72^ ^]^ Clarke used triethylene glycol as a liquid sorbent and used solar distillation technology to desorb water.^[^ ^72^ ^]^

Wang et al. designed a water harvesting device using a CaCl_2_ solution with a mass fraction of 50% as a sorbent, as shown in Figure 7d–f. And, the water production was up to 2.89 kg m^−2^ d^−1^.^[^ ^27^ ^]^ The high‐concentration liquid sorbent is well‐known for high sorption capacity. However, the usage of liquid sorption materials has been primarily limited by the desorption process. As the concentration of liquid sorbent increases, the vaporization enthalpy of liquid sorbent increases, increasing the energy barrier for desorption. It is worth noting that: 1) in the traditional desorption mode driven by sunlight, the liquid sorbents have a low desorption temperature, which makes desorption insufficient or a long desorption time, 2) the recovered liquid water often has residual sorbents, which may be poisonous, and 3) high‐concentration salt solution is prone to corrosion to metal, so it may be limited to some practical application scenarios.

##### Composite Sorbents

The composite sorbents are a kind of hygroscopic water absorbing material with great potential because of its excellent hygroscopic performance, composition adjustability, stability, practicality, low cost, etc.^[^ ^73^, ^74^, ^75^, ^77^ ^]^ Composite sorbents are developed from solid sorbents with advanced performance. They are usually composed of carbon‐based framework with other functional materials. Therefore, the composite sorbents inherit the superior light‐to‐heat conversion performance of carbon‐based solid sorbents. Not only that, this type of composite sorbents also shows excellent adsorption performance due to introduced functional materials and new nanostructure design. Since it can achieve the effective adsorption and desorption processes through the adjustment of components and structure, composite sorbents have shined in recent years.

**Figure 8:**
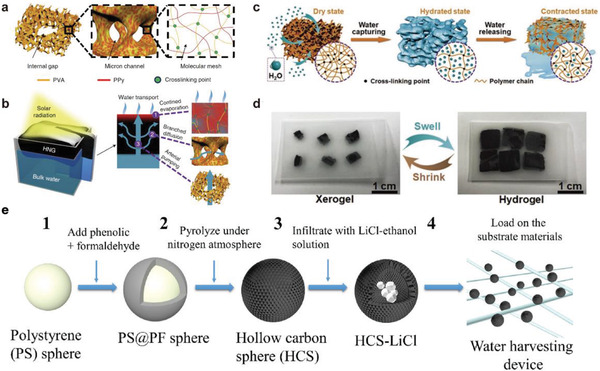
a,b) PVA–PPy composite gel sorbent. Reproduced with permission.^[^ ^76^ ^]^ Copyright 2018, Springer Nature. c,d) PPy–Cl/poly‐NIPAM composite gel sorbent. Reproduced with permission.^[^ ^77^ ^]^ Copyright 2019, John Wiley and Sons. e) hollow carbon spheres (HCS)‐LiCl nanosorbent. Reproduced with permission.^[^ ^78^ ^]^ Copyright 2020, Elsevier.

As early as 1986, Elmer and Hyde proposed a water‐harvesting device made by simply mixing MgCl_2_, LiCl with porous glass, sand, and fiberboard, and found a linear function of the relative humidity to the water adsorption rate.^[^ ^79^ ^]^ In addition, Yu and co‐workers designed a composite sorbent material with a novel gel structure (**Figure** **8**a,b), which was obtained by gelating polypyrrole (PPy) polyvinyl alcohol (PVA) materials. This type of gel sorbent can achieve superhigh water evaporation (≈3.2 kg m^−2^ h^−1^) beyond conventional theoretical limit. They attributed this to the change of structure and interaction force of water molecules, which was verified by the theoretical simulation and thermogravimetric analysis test. Based on this novel system, Yu and co‐workers developed a new composite sorbent, which consists of PPy–Cl and poly‐N‐Isopropylacrylamide (NIPAM) materials at a relative humidity of 60% for 24 h.^[^ ^77^ ^]^ The water adsorption capacity can reach 19.2 kg kg^−1^ (Figure 8c,d).

Wang and co‐workers proposed a composite sorbent composed of polyacrylamide (PAM), carbon nanotubes (CNTs), and calcium chloride (CaCl_2_). The water harvesting device with 35 g of the dry PAM–CNT–CaCl_2_ hydrogel was tested outdoors under field conditions and delivered 20 g of freshwater within 2.5 h under natural sunlight.^[^ ^80^ ^]^ In addition, they explored new composite sorbent with a carbon‐coated LiCl core–shell structure recently.^[^ ^78^ ^]^ At a relative humidity of 60%, it can be adsorbed to a saturation capacity of 1.6 kg kg^−1^ in 3 h. And, due to the carbon‐material‐shell‐induced excellent light absorption and heat conversion efficiency, it can completely desorb the absorbed water within 30 min (Figure 8e).

**Figure 9:**
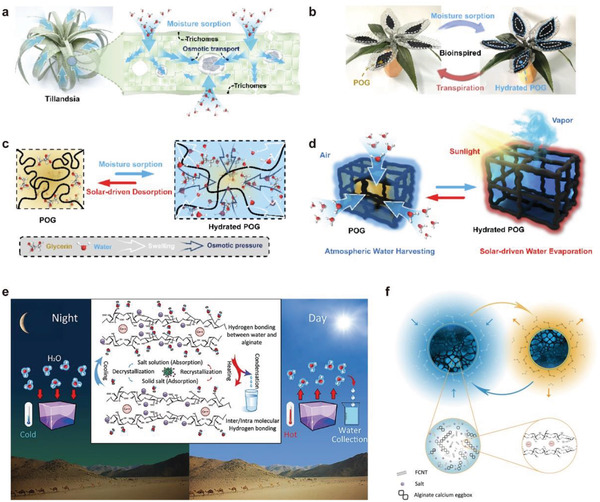
a–d) *Tillandsia*‐inspired hygroscopic photothermal organogels. Reproduced with permission.^[^ ^81^ ^]^ Copyright 2020, John Wiley and Sons. e,f) Superatmospheric water harvesting hydrogel with alginate chains modified with binary salts. Reproduced with permission.^[^ ^82^ ^]^ Copyright 2020, American Chemical Society.

Ni et al. developed an integrated hygroscopic photothermal organogel (POG) to achieve a solar‐powered atmospheric water harvesting inspired by *Tillandsia* (**Figure** **9**a–d).^[^ ^81^ ^]^ Hydrophilic copolymeric frameworks can accommodate hygroscopic glycerin medium, which allows a rapid water diffusion driven by osmotic effect. Thus, POG can quickly reactivate the sorption binding sites for continuous and high adsorption capacity. As a result, the integrated POG sorbent presents the superhigh water adsorption of 16.01 kg m^−2^ at the RH of 90%, and daily water production is 2.43 kg m^−2^ d^−1^ achieved outdoors.

Entezari et al. modified sodium alginate by occupying both G‐blocks and M‐blocks with more hydrophilic cations.^[^ ^82^ ^]^ Functionalized carbon nanotubes (FCNTs) are embedded in the hydrogel structure to increase solar spectrum absorption (Figure 9e,f). The binary composite sorbent can adsorb ≈5.6 g of water per gram of desiccant. Notably, Bina/FCNT is hydrothermally stable and, when employed in a prototype containing 5 g of sorbent, it could produce 1–6 g of water in a still atmosphere, without any air convection or cooling in the condensation process.

In addition, Yao et al. subsequently designed the sodium polyacrylate (PAAS)/graphene framework (PGF) composite gel sorbent to produce clean water from a contaminated atmosphere.^[^ ^83^ ^]^ Therefore, the composite gel sorbents have attracted wide attention because of their excellent adsorption capacity and water production performance.

#### 3.2.2 The Solar‐Driven Photothermal Conversion System

Based on the solar‐driven hygroscopic water harvesting system, solar energy is converted into heat energy to drive the desorption of water in the sorbents. Therefore, the entire system has very high requirements for the photothermal conversion performance of solar energy: 1) broadband and efficient light absorption and 2) high photothermal conversion efficiency of light energy to heat energy.

##### Solar Absorbers

**Figure 10:**
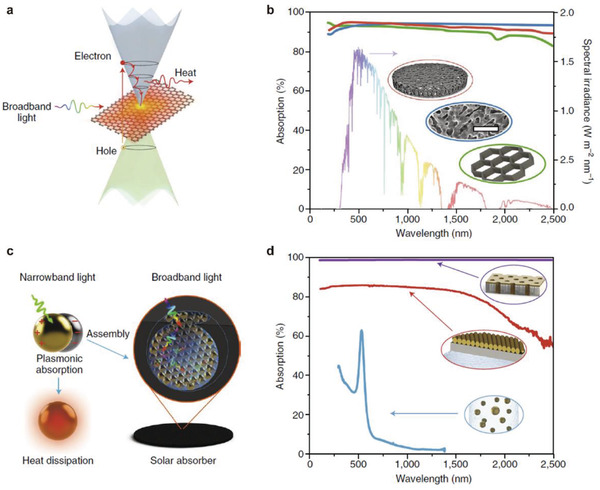
a,b) Carbon‐based absorber materials and c,d) plasmon‐based absorber materials. Reproduced with permission.^[^ ^84^ ^]^ Copyright 2018, Springer Nature.

Broadband and efficient light absorption is a prerequisite for efficient light‐to‐heat conversion. The solar radiation energy is mainly distributed in the range of 280–4000 nm, of which 400–2500 nm accounts for about 99%. An excellent light‐absorbing material needs to reduce transmission and reflection as much as possible in a wide wavelength range to achieve high‐efficiency light absorption.^[^ ^85^, ^86^, ^87^, ^88^, ^89^, ^90^, ^91^, ^92^, ^93^ ^]^ At present, the commonly used broad‐spectrum and efficient light absorber materials mainly include carbon‐based materials and plasmon‐based materials.^[^ ^94^, ^95^, ^96^ ^]^ For light absorption in carbon‐based materials, as shown in **Figure** **10**a,b,^[^ ^97^ ^]^ since Chen and co‐workers reported in 2014 that graphite‐particle absorbers supported on carbon foam have achieved 97% efficient light absorption in 250–2250 nm,^[^ ^98^ ^]^ carbon‐based absorbers have attracted wide attention.^[^ ^99^, ^100^, ^101^, ^102^, ^103^, ^104^, ^105^, ^106^, ^107^, ^108^, ^109^ ^]^ For plasmon‐based materials, as shown in Figure 10c,d,^[^ ^110^, ^111^ ^]^ Zhou et al. designed the self‐assembly absorber with 3D porous anodized aluminum template and gold nanoparticles, and achieved a light absorption efficiency of up to 99% ultrawide bandwidth of 200 nm to 10 µm.^[^ ^112^ ^]^ A series of highly efficient plasmonic photothermal conversion materials have been subsequently reported.^[^ ^113^, ^114^, ^115^, ^116^, ^117^, ^118^ ^]^

##### Photothermal Conversion

As for solid/composite sorbents, carbon‐based or plasmon‐based materials are usually introduced into the system, which can effectively improve the light adsorption and photothermal conversion.

**Figure 11:**
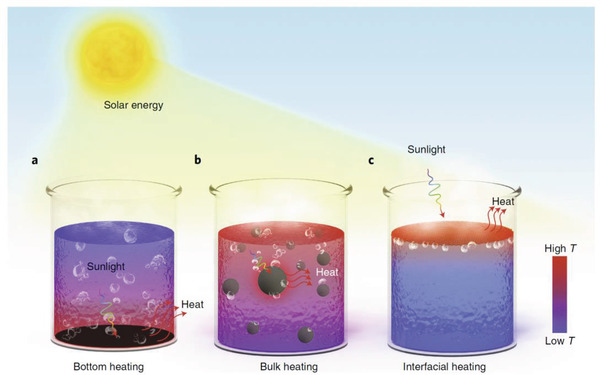
Solar‐driven photothermal liquid water evaporation systems. Water evaporation by a) bottom heating, b) bulk heating, and c) interfacial heating. Reproduced with permission.^[^ ^84^ ^]^ Copyright 2018, Springer Nature.

However, in the liquid sorbent system, its intrinsic light‐to‐heat conversion efficiency is relatively low. Early research was mainly to load the liquid sorbent on the adsorption bed, and convert the light energy into heat energy through the adsorption bed and transfer it to the sorbent to heat it to desorb the water. From initial bottom heating^[^ ^119^ ^]^ to bulk heating,^[^ ^120^, ^121^, ^122^ ^]^ the escape interface of the steam generated moves closer to the interface that converts light into heat, which is interfacial heating (**Figure** **11**). Interfacial heating can achieve solar steam generation efficiency of more than 90% under low‐concentration conditions.^[^ ^98^, ^123^ ^]^ This method selectively heats only the water molecules at the uppermost interface instead of the bulk water, avoiding a large amount of heat loss. It could effectively increase the temperature of the water body at the heated interface, thereby increasing the amount and efficiency of steam generation.^[^ ^66^, ^98^, ^123^, ^124^ ^]^ Due to these advantages, this solar‐driven interface heating technology has been used in water desorption of liquid sorbents, thereby improving the desorption performance of the sorbents. The interfacial solar heating is introduced into the field of atmospheric water harvesting by Wang et al., as shown in Figure 7d–f.^[^ ^27^ ^]^ Interfacial solar heating based on a salt‐resistant graphene oxide (GO)‐based aerogel is shown to enable a high‐concentration liquid‐sorbent (CaCl_2_ 50 wt% solution)‐based atmospheric water generator. Freshwater (2.89 kg m^−2^ d^−1^) can be produced at about 70% relative humidity, with only solar energy input and energy efficiency of desorption as high as 66.9%.

#### 3.2.3 Condensation System

In the design of water harvesting devices, the condensation system is crucial. Under different temperature and relative humidity conditions, the condensation temperature (dew point) of water vapor will change. The driving force to condensation of water vapor is the temperature difference, which drives the condensation and nucleation of gaseous water molecules at the collection interface. Therefore, a sound condensation system needs to have efficient heat exchange performance. In addition, there are certain requirements for the hydrophilicity and hydrophobicity of the condensation surface material.

**Figure 12:**
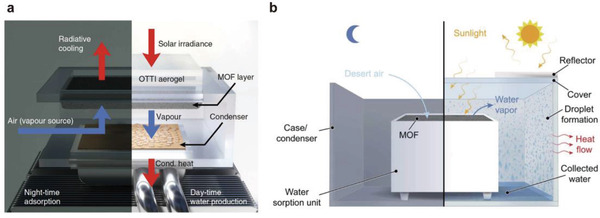
Condensation systems. a) Condensation system of copper tube cooling. Reproduced according to the terms of the CC‐BY license.^[^ ^125^ ^]^ Copyright 2018, Springer Nature. b) Condensation system of high thermal conductivity wall cooling. Reproduced according to the terms of the CC‐BY license.^[^ ^126^ ^]^ Copyright 2018, The American Association for the Advancement of Science.

Kim et al. designed a large copper tube heat exchange component (see **Figure** **12**a) to construct the condensation system, and the environment has efficient heat exchange performance, thereby helping the device promote the condensation of water vapor in a desert environment.^[^ ^125^ ^]^ The condensation wall with a high thermal conductivity was designed by Fathieh et al. to facilitate the condensation process (see Figure 12b).^[^ ^126^ ^]^ In addition, Zhou et al. used radiation cooling materials to promote condensation.^[^ ^127^ ^]^ Regarding the hydrophilicity and hydrophobicity of the condensation surface, Wang and co‐workers studied the heat exchange performance of condensation on the superhydrophobic surface to form droplets and proved that the hydrophobic surface is conducive to the collection of condensed droplets.^[^ ^128^ ^]^

Generally, for the condensation process, it is necessary to continuously optimize the design of the device and design/select materials with excellent heat exchange performance to promote the application of a larger working range of the water‐harvesting device.

### 3.3 Classification of Solar‐Driven Hygroscopic Water‐Harvesting Device

The solar‐driven hygroscopic water‐harvesting device can take the hygroscopic air into the 1) intermittent and 2) sequential types according to the continuity of the water harvesting devices.

#### 3.3.1 Intermittent Solar‐Driven Hygroscopic Water Harvesting

The intermittent water harvesting system is mainly composed of three parts: 1) an adsorption zone, 2) an additional heat removal zone, and 3) a condensation collection zone. According to the structure of the adsorption area, it can be divided into 1) an integrated single‐layer water harvesting structure, 2) multilayer‐type water harvesting structure, and 3) coating‐type structure.

The research on intermittent water‐harvesting devices began in the early 20th century because under natural conditions, the night temperature is low and the relative humidity is high, which is conducive to the capture of water in the air. During the day, the solar heating desorption process takes place. Such related patents were born earlier, and Dunkak designed a simple solar‐powered silicone water‐harvesting device in 1949.^[^ ^131^ ^]^ In the 1990s, Aristov et al. proposed a simple structure of hygroscopic water harvesting.^[^ ^132^ ^]^ Because the difference between its desorption and adsorption mass at 60 °C is only 0.35 g g^−1^, and the total duration of the whole device cycle is 40–80 h, the system is still far from practical application.

##### Single‐Layer Water Harvesting Device

**Figure 13:**
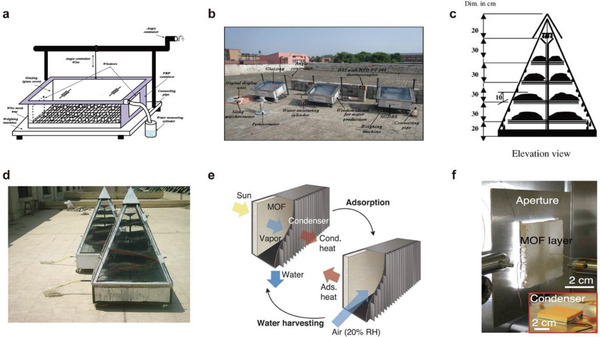
Integrated hygroscopic atmospheric water harvesting system. a,b) Single‐layer water harvesting device. Reproduced with permission.^[^ ^129^ ^]^ Copyright 2015, AIP Publishing. c,d) Multilayer water harvesting device. Reproduced with permission.^[^ ^126^, ^130^ ^]^ Copyright 2007, Elsevier. e,f) Coated water‐harvesting device. Reproduced with permission.^[^ ^67^ ^]^ Copyright 2017, The American Association for the Advancement of Science.

As shown in **Figure** **13**a,b, the working principle of single‐layer water harvesting structure is as follows: place the moisture sorbent at the bottom of the glass box, open the chamber at night, the air contacts the moisture sorbent through natural or forced convection, and the water in the air is captured. During the day, the cavity was closed to seal the entire system, and the temperature of the sorbents was increased under the irradiation of sunlight to heat and desorb the water vapor, and it was collected by condensation at a relatively low‐temperature collection point. This type of water harvesting system is widely used because of its simple structure, low cost, easy maintenance, and small‐scale water harvesting.

Similar work: Hamed and co‐workers used thick corrugated cloth and CaCl_2_ solution as moisture sorbent, with a water production of 1.5 kg m^−2^ d^−1^.^[^ ^133^ ^]^ Ji et al. used MCM‐41/CaCl_2_ material to prepare solar‐powered water‐harvesting device with freshwater generation about 1.2 kg m^−2^ d^−1^.^[^ ^134^ ^]^ However, the shortcomings of this type of device are also revealed. Because of the insufficient condensation by natural convection, the condensed water part will condense on the upper layer of the glass plate, which will affect the light transmittance of the glass and is not conducive to the desorption process. The amount of water generated is about 1–2 kg m^−2^ d^−1^, which is often unable to meet the actual demand. Therefore, in order to increase the water production per unit area, Kabeel studied the water‐harvesting device with a multilayer pyramid structure.^[^ ^130^ ^]^

##### Multilayer Water Harvesting Device

By constructing a multilayer adsorption bed structure, using 30% CaCl_2_ solution as a sorbent, the solar energy is fully utilized to achieve a water production of 2.5 kg m^−2^ d^−1^ (Figure 13c,d).

##### Coated Water‐Harvesting Device

To achieve the performance of water desorption and condensation heat exchange in the sorbents to improve water production efficiency and water production, the coated structure was proposed by researchers. As shown in Figure 13e,f, Kim et al. combined the photothermal conversion material, MOF moisture sorbent, and heat exchanger into one body. During the adsorption process, the air and the MOF material fully contacted, and the water in the air was quickly adsorbed by the moisture sorbent. Under the irradiation of sunlight, the photothermal conversion material converts solar energy into heat energy to heat the sorbents, desorbing the moisture therein; the water vapor contacts the lower temperature heat exchanger to condense and collect liquid water.^[^ ^67^ ^]^ This kind of coating design makes the desorption and condensation process fully enhanced, thereby enhancing the water production and widening the relative humidity range under work.

Intermittent solar‐driven hygroscopic water harvesting is ultimately limited by the amount of water adsorbed at night. During the daylight‐driven desorption process, as the water content in the sorbent continues to decrease, the rate of water desorption decreases sharply, resulting in low efficiency of solar energy utilization and relatively low water production.

#### 3.3.2 Sequential Solar‐Driven Hygroscopic Water Harvesting

Compared with the intermittent water‐harvesting device, the sequential device can make full and more effective use of the limited daylight hours during the day, thereby increasing the daily water production. The sequential solar‐driven hygroscopic water harvesting devices are mainly divided into two types: 1) the rotary solar‐driven atmospheric water‐harvesting devices and 2) interfacial solar‐driven atmospheric water generators with simultaneous adsorption–desorption.

##### The Rotary Solar‐Driven Atmospheric Water‐Harvesting Device

**Figure 14:**
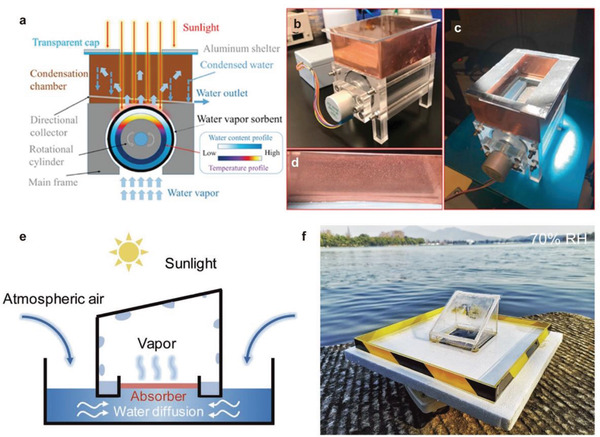
Sequential solar‐driven hygroscopic water harvesting devices. a–d) Rotary atmospheric water‐harvesting device. Reproduced with permission.^[^ ^78^ ^]^ Copyright 2020, Elsevier. e,f) Interfacial solar‐driven atmospheric water generator with simultaneous adsorption–desorption. Reproduced with permission.^[^ ^26^ ^]^ Copyright 2019, John Wiley and Sons.

For the rotary water‐harvesting device, the moisture sorbent is loaded in a rotary structure, and the whole rotary is divided into two areas of adsorption and desorption. In the adsorption area, the sorbents contact the air in the absence of light to absorb moisture in the air; while in the desorption area, the sorbents evaporate the water in the light and then leads it to the condensation area for condensation and collection. Through the rotation of the wheel, the adsorption and desorption areas are constantly switched to achieve continuous water production and maximize the use of limited sunshine time. Rodriguez and Khanji designed an earlier set of rotary dual‐chamber water harvesting device.^[^ ^135^ ^]^ Tongue used the exhaust of the car as a heat source to drive the onboard water harvesting device.^[^ ^136^ ^]^ Milani et al. combined the silicone rotary structure with the fresh air system and constructed the system model using TRNSYS and carried out air water harvesting experiment.^[^ ^137^ ^]^ As shown in **Figure** **14**a–d, Li et al. designed a rotary water‐harvesting device.^[^ ^78^ ^]^ By synthesizing LiCl/C solid sorbents with high water adsorption and fast adsorption performance, and loading them in a rotary structure, the water production of 0.84 g g^−1^ (water/sorbents) under 8 h of light irradiation can be obtained.

##### Interfacial Solar‐Driven Atmospheric Water Generator with Simultaneous Adsorption–Desorption

Incomplete daytime desorption is the limiting factor for final water production, as the rate of water desorption typically decreases very quickly with decreased water content in the sorbents. Qi et al. designed a solar‐driven atmospheric water generator with a simultaneous adsorption–desorption process, combining tailored interfacial solar absorbers with using 1‐ethyl‐3‐methyl‐imidazolium acetate ([EMIM][Ac]) ionic liquid as a liquid sorbent.^[^ ^26^ ^]^ The device can absorb atmospheric water at night, while generating water during the day. It can also continuously absorb water in the air to maintain higher water content in the sorbents, thereby improving the final water collection and energy utilization of the overall device (Figure 14e,f). Moreover, when under 1 sun of irradiation intensity in the lab, the water desorption rate has reached about 0.5 L m^−2^ h^−1^. In a long‐term cycle experiment of 18 h, the entire system shows extremely high stability. The total amount of water collected on an outdoor experiment was as high as 2.8 kg m^−2^ d^−1^.

## 4 Conclusion

At present, the traditional systems of atmospheric water harvesting are based on passive water harvesting or active electric‐driven water harvesting technologies. The applications of these two types of technologies either are limited by climate, weather, or geography or need to consume a large amount of electrical energy. It is significant to meet human demands for freshwater by further utilizing the treasure trove of atmospheric water resources.

Solar‐driven atmospheric water harvesting technology consists of a hygroscopic sorbent as the basis, which can be applied to a wider range of scenarios, and the recycling of the entire system is driven by solar energy. The solar‐powered hygroscopic water‐harvesting device can take the hygroscopic air into the intermittent and sequential types according to the continuity of the water‐harvesting devices.

For the intermittent solar‐driven hygroscopic water harvesting systems, the water in the hygroscopic sorbents cannot be fully evaporated and desorbed during the desorption process, resulting in lower water production. This is due to the poor photothermal conversion performance of the liquid sorbents. During the desorption process, as the concentration of liquid sorbent increases, the vaporization enthalpy of liquid sorbent increases, increasing the energy barrier for desorption. In addition, the intermittent solar‐driven hygroscopic water harvesting system is open at night and absorbs water in the air by the sorbents. The water is desorbed and collected by sunlight during the daytime. Hence, the atmospheric water that can be collected by this type of system is ultimately limited by the amount of water adsorbed at night. During the daylight‐driven desorption process, as the sorbent's water content continues to decrease, the rate of water desorption decreases sharply, resulting in low efficiency of solar energy utilization and relatively low water production.

Compared with the intermittent water‐harvesting devices, the sequential solar‐driven hygroscopic water‐harvesting devices based on the interfacial heating could make much more effective use of the limited solar energy, thereby increasing the daily water production. Based on these characteristics, the sequential solar‐driven hygroscopic water harvesting technology based on the interfacial heating is expected to be used for freshwater production, particularly for areas that lack direct access to clean water. Additionally, due to the high photothermal conversion efficiency, interfacial heating is promising to be widely used in other fields, such as solar desalination,^[^ ^101^, ^108^, ^124^, ^138^, ^139^, ^140^, ^141^, ^142^, ^143^, ^144^ ^]^ wastewater treatment,^[^ ^66^, ^76^, ^98^, ^99^, ^104^, ^112^, ^113^, ^116^, ^118^, ^122^, ^145^, ^146^, ^147^, ^148^, ^149^, ^150^, ^151^, ^152^ ^]^ high‐salinity brine treatment,^[^ ^153^ ^]^ power generation,^[^ ^154^, ^155^, ^156^ ^]^ synergistic solar electricity‐water generation,^[^ ^157^ ^]^ etc.

To establish an effective, scalable, and low‐cost method for collecting atmospheric water, the aspects that may need in‐depth thinking and exploration for the solar‐driven hygroscopic water harvesting in the future research are as follows: 1) microscopic mechanism of adsorption, transport, and desorption of water vapor in the hygroscopic agents, 2) development or modification of the new types of hygroscopic agents for sequential‐type solar‐driven hygroscopic water‐harvesting devices, 3) modification of the light absorber's microstructure to reduce the enthalpy of evaporation to further improve the water production, photothermal conversion efficiency, and device stability, 4) design of multilayered condensation system and utilization of more efficient heat exchange components for improving the condensation efficiency of the device and increasing water production per unit area per day.

## Conflict of Interest

The authors declare no conflict of interest.

## References

[1] C. Chen , Y. Kuang , L. Hu , Joule 2019, 3, 683.

[2] M. M. Mekonnen , A. Y. Hoekstra , Sci. Adv. 2016, 2, e1500323. 26933676 10.1126/sciadv.1500323 PMC4758739

[3] S. Neto , in Water Challenges of an Urbanizing World (Ed: Glavan M.), IntechOpen, London 2018, Ch. 6.

[4] P. Gleick , in Encyclopedia of Climate and Weather, Vol. 2 (Eds: Schneider S., Mastrandrea M. D., Root T. L.), Oxford University Press, New York 1996, p. 817.

[5] A. E. Ercin , A. Y. Hoekstra , Environ. Int. 2014, 64, 71. 24374780 10.1016/j.envint.2013.11.019

[6] M. Elimelech , W. A. Phillip , Science 2011, 333, 712. 21817042 10.1126/science.1200488

[7] C. Li , D. Goswami , E. K. Stefanakos , Renewable Sustainable Energy Rev. 2013, 19, 136.

[8] H. Sharon , K. S. Reddy , Renewable Sustainable Energy Rev. 2015, 41, 1080.

[9] D. Cohen‐Tanugi , J. C. Grossman , Nano Lett. 2012, 12, 3602. 22668008 10.1021/nl3012853

[10] S. A. Kalogirou , Prog. Energy Combust. Sci. 2005, 31, 242.

[11] A. Pugsley , A. Zacharopoulos , J. D. Mondol , M. Smyth , Renewable Energy 2016, 88, 200.

[12] Office‐of‐Water , Office‐of‐Wastewater‐Management , Primer for Municipal Wastewater Treatment Systems, U.S. Environmental Protection Agency, Washington, D.C., USA 2004, EPA 832‐R‐04‐001.

[13] D. Beysens , Atmos. Res. 2016, 167, 146.

[14] D. Beysens , I. Milimouk , A. Schweitzer , Sécheresse 2000, 11, 281.

[15] P. H. Gleick , in The World's Water (Eds: Gleick P. H., Ajami N., Christian‐Smith J., Cooley H., Donnelly K., Fulton J., Ha M.‐L., Heberger M., Moore E., Morrison J., Orr S., Schulte P., Srinivasan V.), Island Press, Washington, D.C., USA 2018.

[16] V. S. Nikolayev , D. Beysens , A. Gioda , I. Milimouka , E. Katiushin , J. P. Morel , J. Hydrol. 1996, 182, 19.

[17] R. S. Schemenauer , P. Cereceda , Ambio 1991, 20, 303.

[18] J. L. Monteith , Q. J. R. Meteorol. Soc. 1957, 83, 322.

[19] I. Gindel , Nature 1965, 207, 1173.

[20] B. Khalil , J. Adamowski , A. Shabbir , C. Jang , M. Rojas , K. Reilly , B. Ozga‐Zielinski , Sustainable Water Resour. Manage. 2016, 2, 71.

[21] Y. D. Tu , R. Z. Wang , Y. N. Zhang , J. Y. Wang , Joule 2018, 2, 1452.

[22] G. M. Peters , N. J. Blackburn , M. Armedion , Int. J. Life Cycle Assess. 2013, 18, 1149.

[23] M. Fessehaye , S. A. Abdul‐Wahab , M. J. Savage , T. Kohler , T. Gherezghiher , H. Hurni , Renewable Sustainable Energy Rev. 2014, 29, 52.

[24] R. V. Wahlgren , Water Res. 2001, 35, 1. 11257862 10.1016/s0043-1354(00)00247-5

[25] D. Bergmair , S. J. Metz , H. C. de Lange , A. A. van Steenhoven , Desalination 2014, 339, 26.

[26] H. Qi , T. Wei , W. Zhao , B. Zhu , G. Liu , P. Wang , Z. Lin , X. Wang , X. Li , X. Zhang , J. Zhu , Adv. Mater. 2019, 31, 1903378. 10.1002/adma.201903378 31523873

[27] X. Wang , X. Li , G. Liu , J. Li , X. Hu , N. Xu , W. Zhao , B. Zhu , J. Zhu , Angew. Chem., Int. Ed. 2019, 58, 12054. 10.1002/anie.201905229 31197935

[28] Wet Pipe Potable Rainwater Collection System, Innovative Water Solution LLC, Austin, TX.

[29] K.‐C. Park , S. S. Chhatre , S. Srinivasan , R. E. Cohen , G. H. McKinley , Langmuir 2013, 29, 13269. 23895249 10.1021/la402409f

[30] W. Shi , M. J. Anderson , J. B. Tulkoff , B. S. Kennedy , J. B. Boreyko , ACS Appl. Mater. Interfaces 2018, 10, 11979. 29587482 10.1021/acsami.7b17488

[31] Y. Tian , P. G. Zhu , X. Tang , C. M. Zhou , J. M. Wang , T. T. Kong , M. Xu , L. Q. Wang , Nat. Commun. 2017, 8, 15823. 28604698 10.1038/ncomms15823 PMC5472779

[32] D. Beysens , I. Milimouk , V. Nikolayev , M. Muselli , J. Marcillat , J. Hydrol. 2003, 276, 1.

[33] A. Gu , *Master Thesis*, Zhengzhou University 2017.

[34] I. Gultepe , R. Tardif , S. C. Michaelides , J. Cermak , A. Bott , J. Bendix , M. D. Müller , M. Pagowski , B. Hansen , G. Ellrod , W. Jacobs , G. Toth , S. G. Cober , Pure Appl. Geophys. 2007, 164, 1121.

[35] A. F. Batisha , Sustainability Water Qual. Ecol. 2015, 6, 1.

[36] S. Montecinos , D. Carvajal , P. Cereceda , M. Concha , Atmos. Res. 2018, 209, 163.

[37] S. A. Abdul‐Wahab , H. Al‐Hinai , K. A. Al‐Najar , M. S. Al‐Kalbani , J. Water Supply: Res. Technol.–AQUA 2007, 56, 275.

[38] E. S. Shanyengana , P. Cereceda , P. Osses , presented at XIth IRCSA Conf., Texcoco, Mexico, August 2003, p. 20.

[39] G. Sharan , presented at Int. Conf. on Agricultural Engineering, Hersonissos, Crete, Greece, June 2008, pp. 23–25.

[40] G. Sharan , A. K. Roy , L. Royon , A. Mongruel , D. Beysens , J. Cleaner Prod. 2017, 155, 83.

[41] B. Kotzen , presented at EGU General Assembly Conf., Vienna, Austria, April to May, 2014.

[42] M. A. Munoz‐Garcia , G. P. Moreda , M. P. Raga‐Arroyo , O. Marin‐Gonzalez , Comput. Electron. Agric. 2013, 93, 60.

[43] A. P. Raman , M. A. Anoma , L. Zhu , E. Rephaeli , S. Fan , Nature 2014, 515, 540. 25428501 10.1038/nature13883

[44] Y. Zhai , Y. Ma , S. N. David , D. Zhao , R. Lou , G. Tan , R. Yang , X. Yin , Science 2017, 355, 1062. 28183998 10.1126/science.aai7899

[45] R. H. Qi , D. J. Li , L. Z. Zhang , Appl. Energy 2017, 208, 1174.

[46] D. Bergmair , S. J. Metz , H. C. de Lange , A. A. van Steenhoven , Sep. Purif. Technol. 2015, 150, 112.

[47] G. G. Bernard , *US2409624A*, 1946.

[48] A. A. Al‐Farayedhi , N. I. Ibrahim , P. Gandhidasan , Desalination 2014, 349, 60.

[49] B. Khalil , J. Adamowski , M. Rojas , K. Reilly , presented at American Society of Agricultural and Biological Engineers Int. Meeting, Montreal, 13–16 July, 2014.

[50] D. T. Bui , K. J. Chua , J. M. Gordon , Science 2017, 358, eaao0791. 29191876 10.1126/science.aao0791

[51] M. J. Moran , H. N. Shapiro , J. Therm. Anal. Calorim. 2004, 60, 707.

[52] L. Zoontjens , C. Q. Howard , A. Zander , B. S. Cazzolato , Proc. Acoust. 2005.

[53] K. A. Gschneidner , A. O. Pecharsky , D. Jiles , Off. Sci. Tech. Inf. Tech. Rep. 2001.

[54] S. K. Fischer , Off. Sci. Tech. Inf. Tech. Rep. 2001.

[55] D. Butler , CIBSE National Conf., 2001.

[56] M. Tircot , *US4506510A*, London, UK, November 2001.

[57] J. C. Peltier , Ann. Chim. 1834, 56, 371.

[58] Y. L. Wu , X. Peng , J. Z. Liu , Q. Y. Kong , B. L. Shi , M. S. Tong , J. Membr. Sci. 2002, 196, 179.

[59] H. Sijbesma , K. Nymeijer , R. van Marwijk , R. Heijboer , J. Potreck , M. Wessling , J. Membr. Sci. 2008, 313, 263.

[60] L. Liu , Y. Chen , Y. X. Kang , M. Deng , Chem. Eng. Technol. 2001, 24, 1045.

[61] S. C. George , S. Thomas , Prog. Polym. Sci. 2001, 26, 985.

[62] B. Gebben , J. Membr. Sci. 1996, 113, 323.

[63] Solargis Imaps Global Horizontal Solar Irradiations , https://solargis.com/maps‐and‐gis‐data/download/world (accessed: November 2020).

[64] M. M. Gao , L. L. Zhu , C. K. Peh , G. W. Ho , Energy Environ. Sci. 2019, 12, 841.

[65] C. Jia , Y. Li , Z. Yang , G. Chen , Y. Yao , F. Jiang , Y. Kuang , G. Pastel , H. Xie , B. Yang , S. Das , L. Hu , Joule 2017, 1, 588.

[66] G. Ni , G. Li , S. V. Boriskina , H. Li , W. Yang , T. Zhang , G. Chen , Nat. Energy 2016, 1, 16126.

[67] H. Kim , S. Yang , S. R. Rao , S. Narayanan , E. A. Kapustin , H. Furukawa , A. S. Umans , O. M. Yaghi , E. N. Wang , Science 2017, 356, 430. 28408720 10.1126/science.aam8743

[68] X. Zheng , T. S. Ge , R. Z. Wang , Energy 2014, 74, 280.

[69] Y. Kong , X. D. Shen , S. Cui , M. H. Fan , Appl. Energy 2015, 147, 308.

[70] S. T. Wilson , B. M. Lok , C. A. Messina , T. R. Cannan , E. M. Flanigen , J. Am. Chem. Soc. 1982, 104, 1146.

[71] B. Lund , *US3777456*, 1973.

[72] N. P. Clarke , *US5233843A*, 1993.

[73] J. Y. Liu , J. Y. Wang , L. W. Wang , R. Z. Wang , Int. J. Refrig. 2017, 83, 51.

[74] I. A. Simonova , A. Freni , G. Restuccia , Y. I. Aristov , Microporous Mesoporous Mater. 2009, 122, 223.

[75] N. Yu , R. Z. Wang , Z. S. Lu , L. W. Wang , Chem. Eng. Sci. 2014, 111, 73.

[76] F. Zhao , X. Zhou , Y. Shi , X. Qian , M. Alexander , X. Zhao , S. Mendez , R. Yang , L. Qu , G. Yu , Nat. Nanotechnol. 2018, 13, 489. 29610528 10.1038/s41565-018-0097-z

[77] F. Zhao , X. Zhou , Y. Liu , Y. Shi , Y. Dai , G. Yu , Adv. Mater. 2019, 31, 1806446. 10.1002/adma.201806446 30633394

[78] R. Li , Y. Shi , M. Wu , S. Hong , P. Wang , Nano Energy 2020, 67, 104255.

[79] T. H. Elmer , J. F. Hyde , Sep. Sci. Technol. 1986, 21, 251.

[80] R. Li , Y. Shi , M. Alsaedi , M. Wu , L. Shi , P. Wang , Environ. Sci. Technol. 2018, 52, 11367. 30192516 10.1021/acs.est.8b02852

[81] F. Ni , N. Qiu , P. Xiao , C. Zhang , Y. Jian , Y. Liang , W. Xie , L. Yan , T. Chen , Angew. Chem., Int. Ed. 2020, 59, 19237. 10.1002/anie.202007885 33448559

[82] A. Entezari , M. Ejeian , R. Wang , ACS Mater. Lett. 2020, 2, 471.

[83] H. Yao , P. Zhang , Y. Huang , H. Cheng , C. Li , L. Qu , Adv. Mater. 2020, 32, 1905875. 10.1002/adma.201905875 31856369

[84] P. Tao , G. Ni , C. Song , W. Shang , J. Wu , J. Zhu , G. Chen , T. Deng , Nat. Energy 2018, 3, 1031.

[85] Y. Wang , F. Zhang , X. Tang , X. Chen , Y. Chen , W. Huang , Z. Liang , L. Wu , Y. Ge , Y. Song , J. Liu , D. Zhang , J. Li , H. Zhang , Laser Photonics Rev. 2018, 12, 1800016.

[86] Q. Wang , Y. Chen , L. Miao , G. Jiang , S. Chen , J. Liu , X. Fu , C. Zhao , H. Zhang , Opt. Express 2015, 23, 7681. 25837106 10.1364/OE.23.007681

[87] Q. Ou , Y. Zhang , Z. Wang , J. A. Yuwono , R. Wang , Z. Dai , W. Li , C. Zheng , Z. Q. Xu , X. Qi , S. Duhm , N. V. Medhekar , H. Zhang , Q. Bao , Adv. Mater. 2018, 30, 1705792. 10.1002/adma.201705792 29493028

[88] C. Xing , W. Huang , Z. Xie , J. Zhao , D. Ma , T. Fan , W. Liang , Y. Ge , B. Dong , J. Li , H. Zhang , ACS Photonics 2017, 5, 621.

[89] L. Wu , Z. Xie , L. Lu , J. Zhao , Y. Wang , X. Jiang , Y. Ge , F. Zhang , S. Lu , Z. Guo , J. Liu , Y. Xiang , S. Xu , J. Li , D. Fan , H. Zhang , Adv. Opt. Mater. 2018, 6, 1700985.

[90] T. T. Lv , Y. X. Li , H. F. Ma , Z. Zhu , Z. P. Li , C. Y. Guan , J. H. Shi , H. Zhang , T. J. Cui , Sci. Rep. 2016, 6, 23186. 27000427 10.1038/srep23186 PMC4802382

[91] Z. Chu , J. Liu , Z. Guo , H. Zhang , Opt. Mater. Express 2016, 6, 2374.

[92] J. Du , M. Zhang , Z. Guo , J. Chen , X. Zhu , G. Hu , P. Peng , Z. Zheng , H. Zhang , Sci. Rep. 2017, 7, 42357. 28211471 10.1038/srep42357 PMC5314455

[93] J. Zheng , X. Tang , Z. Yang , Z. Liang , Y. Chen , K. Wang , Y. Song , Y. Zhang , J. Ji , Y. Liu , D. Fan , H. Zhang , Adv. Opt. Mater. 2017, 5, 1700026.

[94] J. Shao , L. Tong , S. Tang , Z. Guo , H. Zhang , P. Li , H. Wang , C. Du , X. F. Yu , ACS Appl. Mater. Interfaces 2015, 7, 5391. 25697378 10.1021/am508881k

[95] Z. Zhang , Y. Liu , L. Ren , H. Zhang , Z. Huang , X. Qi , X. Wei , J. Zhong , Electrochim. Acta 2016, 200, 142.

[96] Z. Huang , Z. Zhang , X. Qi , X. Ren , G. Xu , P. Wan , X. Sun , H. Zhang , Nanoscale 2016, 8, 13273. 27336591 10.1039/c6nr04020a

[97] A. B. Kuzmenko , E. van Heumen , F. Carbone , D. van der Marel , Phys. Rev. Lett. 2008, 100, 117401. 18517825 10.1103/PhysRevLett.100.117401

[98] H. Ghasemi , G. Ni , A. M. Marconnet , J. Loomis , S. Yerci , N. Miljkovic , G. Chen , Nat. Commun. 2014, 5, 4449. 25043613 10.1038/ncomms5449

[99] Y. F. Song , H. Zhang , D. Y. Tang , D. Y. Shen , Opt. Express 2012, 20, 27283. 23187583 10.1364/OE.20.027283

[100] Y. Ito , Y. Tanabe , J. Han , T. Fujita , K. Tanigaki , M. Chen , Adv. Mater. 2015, 27, 4302. 26079440 10.1002/adma.201501832

[101] Z. Huang , W. Han , H. Tang , L. Ren , D. S. Chander , X. Qi , H. Zhang , 2D Mater. 2015, 2, 035011.

[102] X. Li , W. Xu , M. Tang , L. Zhou , B. Zhu , S. Zhu , J. Zhu , Proc. Natl. Acad. Sci. USA 2016, 113, 13953. 27872280 10.1073/pnas.1613031113 PMC5150409

[103] X. Z. Wang , Y. R. He , G. Cheng , L. Shi , X. Liu , J. Q. Zhu , Energy Convers. Manage. 2016, 130, 176.

[104] X. Hu , W. Xu , L. Zhou , Y. Tan , Y. Wang , S. Zhu , J. Zhu , Adv. Mater. 2017, 29, 1604031. 10.1002/adma.201604031 27885728

[105] P. H. Yang , K. Liu , Q. Chen , J. Li , J. J. Duan , G. B. Xue , Z. S. Xu , W. K. Xie , J. Zhou , Energy Environ. Sci. 2017, 10, 1923.

[106] Z. Yin , H. M. Wang , M. Q. Jian , Y. S. Li , K. L. Xia , M. C. Zhang , C. Y. Wang , Q. Wang , M. Ma , Q. S. Zheng , Y. Y. Zhang , ACS Appl. Mater. Interfaces 2017, 9, 28596. 28772073 10.1021/acsami.7b08619

[107] Y. J. Li , T. T. Gao , Z. Yang , C. J. Chen , W. Luo , J. W. Song , E. Hitz , C. Jia , Y. B. Zhou , B. Y. Liu , B. Yang , L. B. Hu , Adv. Mater. 2017, 29, 1700981. 10.1002/adma.201700981 28470982

[108] N. Xu , X. Hu , W. Xu , X. Li , L. Zhou , S. Zhu , J. Zhu , Adv. Mater. 2017, 29, 1606762. 10.1002/adma.201606762 28520092

[109] S. Bai , C. Sun , H. Yan , X. Sun , H. Zhang , L. Luo , X. Lei , P. Wan , X. Chen , Small 2015, 11, 5807. 26395971 10.1002/smll.201502169

[110] M. L. Brongersma , N. J. Halas , P. Nordlander , Nat. Nanotechnol. 2015, 10, 25. 25559968 10.1038/nnano.2014.311

[111] H. H. Richardson , M. T. Carlson , P. J. Tandler , P. Hernandez , A. O. Govorov , Nano Lett. 2009, 9, 1139. 19193041 10.1021/nl8036905 PMC2669497

[112] L. Zhou , Y. Tan , D. Ji , B. Zhu , P. Zhang , J. Xu , Q. Gan , Z. Yu , J. Zhu , Sci. Adv. 2016, 2, e1501227. 27152335 10.1126/sciadv.1501227 PMC4846456

[113] L. Zhou , S. Zhuang , C. He , Y. Tan , Z. Wang , J. Zhu , Nano Energy 2017, 32, 195.

[114] X. Wang , Y. He , X. Liu , G. Cheng , J. Zhu , Appl. Energy 2017, 195, 414.

[115] L. L. Zhang , J. Xing , X. L. Wen , J. W. Chai , S. J. Wang , Q. H. Xiong , Nanoscale 2017, 9, 12843. 28832043 10.1039/c7nr05149b

[116] Y. Liu , S. Yu , R. Feng , A. Bernard , Y. Liu , Y. Zhang , H. Duan , W. Shang , P. Tao , C. Song , T. Deng , Adv. Mater. 2015, 27, 2768. 25809733 10.1002/adma.201500135

[117] J. Fang , Q. L. Liu , W. Zhang , J. J. Gu , Y. S. Su , H. L. Su , C. P. Guo , D. Zhang , J. Mater. Chem. A 2017, 5, 17817.

[118] K. Bae , G. Kang , S. K. Cho , W. Park , K. Kim , W. J. Padilla , Nat. Commun. 2015, 6, 10103. 26657535 10.1038/ncomms10103 PMC4682046

[119] A. E. Kabeel , S. A. El‐Agouz , Desalination 2011, 276, 1.

[120] G. Ni , N. Miljkovic , H. Ghasemi , X. Huang , S. V. Boriskina , C.‐T. Lin , J. Wang , Y. Xu , M. M. Rahman , T. Zhang , G. Chen , Nano Energy 2015, 17, 290.

[121] H. C. Jin , G. P. Lin , L. Z. Bai , A. Zeiny , D. S. Wen , Nano Energy 2016, 28, 397.

[122] O. Neumann , A. S. Urban , J. Day , S. Lal , P. Nordlander , N. J. Halas , ACS Nano 2013, 7, 42. 23157159 10.1021/nn304948h

[123] Z. Wang , Y. Liu , P. Tao , Q. Shen , N. Yi , F. Zhang , Q. Liu , C. Song , D. Zhang , W. Shang , T. Deng , Small 2014, 10, 3234. 24821378 10.1002/smll.201401071

[124] L. Zhou , Y. Tan , J. Wang , W. Xu , Y. Yuan , W. Cai , S. Zhu , J. Zhu , Nat. Photonics 2016, 10, 393.

[125] H. Kim , S. R. Rao , E. A. Kapustin , L. Zhao , S. Yang , O. M. Yaghi , E. N. Wang , Nat. Commun. 2018, 9, 1191. 29568033 10.1038/s41467-018-03162-7 PMC5864962

[126] F. Fathieh , M. J. Kalmutzki , E. A. Kapustin , P. J. Waller , J. Yang , O. M. Yaghi , Sci. Adv. 2018, 4, eaat3198. 29888332 10.1126/sciadv.aat3198 PMC5993474

[127] M. Zhou , H. Song , X. Xu , A. Shahsafi , Z. Xia , Z. Ma , M. A. Kats , J. Zhu , B. S. Ooi , Q. Gan , Z. Yu , in New Concepts in Solar and Thermal Radiation Conversion II, International Society for Optics and Photonics, 2019, vol. 11121, P. 1112107.

[128] N. Miljkovic , R. Enright , E. N. Wang , ACS Nano 2012, 6, 1776. 22293016 10.1021/nn205052a

[129] M. Kumar , A. Yadav , J. Renewable Sustainable Energy 2015, 7, 033122.

[130] A. E. Kabeel , Renewable Energy 2007, 32, 157.

[131] E. B. Dunkak , *US2462952*, 1949.

[132] Y. I. Aristov , M. M. Tokarev , L. G. Gordeeva , V. N. Snytnikov , V. N. Parmon , Sol. Energy 1999, 66, 165.

[133] H. E. Gad , A. M. Hamed , I. I. El‐Sharkawy , Renewable Energy 2001, 22, 541.

[134] J. G. Ji , R. Z. Wang , L. X. Li , Desalination 2007, 212, 176.

[135] F. Rodriguez , N. K. Khanji , *US8118912B2*, 2012.

[136] S. Tongue , *US7251945B2*, 2007.

[137] D. Milani , A. Qadir , A. Vassallo , M. Chiesa , A. Abbas , Energy Build. 2014, 77, 236.

[138] X. Li , G. Ni , T. Cooper , N. Xu , J. Li , L. Zhou , X. Hu , B. Zhu , P. Yao , J. Zhu , Joule 2019, 3, 1798.

[139] X. Hu , J. Zhu , Adv. Funct. Mater. 2020, 30, 1907234.

[140] W. Xu , X. Hu , S. Zhuang , Y. Wang , X. Li , L. Zhou , S. Zhu , J. Zhu , Adv. Energy Mater. 2018, 8, 1702884.

[141] M. Gao , P. K. N. Connor , G. W. Ho , Energy Environ. Sci. 2016, 9, 3151.

[142] X. Wang , G. Ou , N. Wang , H. Wu , ACS Appl. Mater. Interfaces 2016, 8, 9194. 27019007 10.1021/acsami.6b02071

[143] P. Zhang , Q. Liao , H. Yao , Y. Huang , H. Cheng , L. Qu , Energy Storage Mater. 2019, 18, 429.

[144] H. Geng , Q. Xu , M. Wu , H. Ma , P. Zhang , T. Gao , L. Qu , T. Ma , C. Li , Nat. Commun. 2019, 10, 1512. 30944322 10.1038/s41467-019-09535-w PMC6447597

[145] J. Li , X. Wang , Z. Lin , N. Xu , X. Li , J. Liang , W. Zhao , R. Lin , B. Zhu , G. Liu , L. Zhou , S. Zhu , J. Zhu , Joule 2020, 4, 928.

[146] X. Li , J. Li , J. Lu , N. Xu , C. Chen , X. Min , B. Zhu , H. Li , L. Zhou , S. Zhu , T. Zhang , J. Zhu , Joule 2018, 2, 1331.

[147] M. Jiang , Q. Shen , J. Zhang , S. An , S. Ma , P. Tao , C. Song , B. Fu , J. Wang , T. Deng , W. Shang , Adv. Funct. Mater. 2020, 30, 1910481.

[148] Y. Shi , R. Li , Y. Jin , S. Zhuo , L. Shi , J. Chang , S. Hong , K.‐C. Ng , P. Wang , Joule 2018, 2, 1171.

[149] T. A. Cooper , S. H. Zandavi , G. W. Ni , Y. Tsurimaki , Y. Huang , S. V. Boriskina , G. Chen , Nat. Commun. 2018, 9, 5086. 30538234 10.1038/s41467-018-07494-2 PMC6290071

[150] E. Chiavazzo , M. Morciano , F. Viglino , M. Fasano , P. Asinari , Nat. Sustainability 2018, 1, 763.

[151] L. Zhang , B. Tang , J. Wu , R. Li , P. Wang , Adv. Mater. 2015, 27, 4889. 26184454 10.1002/adma.201502362

[152] M. S. Zielinski , J. W. Choi , T. La Grange , M. Modestino , S. M. Hashemi , Y. Pu , S. Birkhold , J. A. Hubbell , D. Psaltis , Nano Lett. 2016, 16, 2159. 26918518 10.1021/acs.nanolett.5b03901

[153] N. Xu , J. Li , Y. Wang , C. Fang , X. Li , Y. Wang , L. Zhou , B. Zhu , Z. Wu , S. Zhu , J. Zhu , Sci. Adv. 2019, 5, eaaw7013. 31281896 10.1126/sciadv.aaw7013 PMC6611683

[154] X. Li , X. Min , J. Li , N. Xu , P. Zhu , B. Zhu , S. Zhu , J. Zhu , Joule 2018, 2, 2477.

[155] X. Chen , D. Goodnight , Z. Gao , A. H. Cavusoglu , N. Sabharwal , M. DeLay , A. Driks , O. Sahin , Nat. Commun. 2015, 6, 7346. 26079632 10.1038/ncomms8346 PMC4490384

[156] F. Gao , W. Li , X. Wang , X. Fang , M. Ma , Nano Energy 2016, 22, 19.

[157] N. Xu , P. Zhu , Y. Sheng , L. Zhou , X. Li , H. Tan , S. Zhu , J. Zhu , Joule 2020, 4, 347.

